# A Novel Strategy for Creating Tissue-Engineered Biomimetic Blood Vessels Using 3D Bioprinting Technology

**DOI:** 10.3390/ma11091581

**Published:** 2018-09-01

**Authors:** Yuanyuan Xu, Yingying Hu, Changyong Liu, Hongyi Yao, Boxun Liu, Shengli Mi

**Affiliations:** 1 Biomanufacturing and Rapid Forming Technology Key Laboratory of Beijing, Department of Mechanical Engineering, Tsinghua University, Beijing 100084, China; xuyy16@sz.tsinghua.edu.cn; 2 Biomanufacturing Engineering Laboratory, Advanced Manufacturing Division, Graduate School at Shenzhen, Tsinghua University, Shenzhen 518055, China; huyy15@mails.tsinghua.edu.cn (Y.H.); yaohy16@mails.tsinghua.edu.cn (H.Y.); 3 Additive Manufacturing Research Institute, College of Mechatronics and Control Engineering, Shenzhen University, Shenzhen 518060, China; liuchangyong@aliyun.com; 4 Department of Precision Medicine and Healthcare, Tsinghua-Berkeley Shenzhen Institute, Shenzhen 518055, China; lbx16@mails.tsinghua.edu.cn; 5 Open FIESTA Center, Tsinghua University, Shenzhen 518055, China

**Keywords:** 3D bioprinting, vascularized tissues, small-diameter blood vessels, biomimetic modeling, dECM

## Abstract

In this work, a novel strategy was developed to fabricate prevascularized cell-layer blood vessels in thick tissues and small-diameter blood vessel substitutes using three-dimensional (3D) bioprinting technology. These thick vascularized tissues were comprised of cells, a decellularized extracellular matrix (dECM), and a vasculature of multilevel sizes and multibranch architectures. Pluronic F127 (PF 127) was used as a sacrificial material for the formation of the vasculature through a multi-nozzle 3D bioprinting system. After printing, Pluronic F127 was removed to obtain multilevel hollow channels for the attachment of human umbilical vein endothelial cells (HUVECs). To reconstruct functional small-diameter blood vessel substitutes, a supporting scaffold (SE1700) with a double-layer circular structure was first bioprinted. Human aortic vascular smooth muscle cells (HA-VSMCs), HUVECs, and human dermal fibroblasts–neonatal (HDF-n) were separately used to form the media, intima, and adventitia through perfusion into the corresponding location of the supporting scaffold. In particular, the dECM was used as the matrix of the small-diameter blood vessel substitutes. After culture in vitro for 48 h, fluorescent images revealed that cells maintained their viability and that the samples maintained structural integrity. In addition, we analyzed the mechanical properties of the printed scaffold and found that its elastic modulus approximated that of the natural aorta. These findings demonstrate the feasibility of fabricating different kinds of vessels to imitate the structure and function of the human vascular system using 3D bioprinting technology.

## 1. Introduction

Vascular diseases have recently become an important threat to human health and, at present, are mainly treated with vascular grafts. Traditional grafting methods are limited by the source, low long-term patency, and mismatching with natural vascular compliance [1]. With the development of tissue engineering, three-dimensional (3D) bioprinting has emerged as a promising method to biofabricate biomimetic blood vessels [2,3,4,5,6,7]. The introduction of 3D printing and textile techniques to fabricate vascularized tissues and small-diameter blood vessels has also opened several new and exciting avenues in the areas of vascular engineering and regenerative medicine [8,9,10,11,12,13]. However, there are still some requirements to be met for the 3D bioprinting process, such as biocompatibility (suitable for cell growth, migration, and reproduction) and mechanical properties (replicating in vivo architectural features), with the aim of constructing biomimetic blood vessels [14,15,16,17]. 

Vascular vessels include arteries, veins, and capillaries [18]. On the one hand, for capillary structure, common manufacturing methods in vitro mainly include three methods: the removal of sacrificial materials, induced formation, and direct construction [19,20,21]. Growth factor-induced angiogenesis in vivo is a principle-based approach with a very strong bionic foundation and many induced uncontrollable and uncertain factors [22,23]. Direct formation has the advantage of avoiding the introduction of other materials and preventing contact with any cytotoxic substances, but the disadvantage is a lack of high precision and difficult operation [24]. The sacrificial method is amenable to the use of a very wide range of materials. It is easy to operate and can be applied flexibly to hierarchical structures (channels with different diameters) [25]. It can even be used for simple branching structures [26]. The limitations of this approach are that the matrix material and vascular channels cannot be formed simultaneously and that it is difficult to control the accurate distribution of the cells in thick tissues. On the other hand, arteriovenous structures such as small-diameter blood vessels consist of three layers of membrane structures, namely, the adventitia, media, and intima [27]. Constructing a three-tunic structure of vessels becomes possible after years of development. For example, using a rotary printing method, Gao et al. bioprinted vessel-like structures with multilevel fluidic channels, which have potential applications in organ-on-chip devices [28]. The limitation of this method is that it does not accurately imitate the three-layer spatial characteristics of blood vessels in vitro. Schöneberg et al. used a drop-on-demand bioprinting technique to generate in vitro blood vessel models, consisting of a continuous endothelium imitating the tunica intima, an elastic smooth muscle cell layer mimicking the tunica media, and a surrounding fibrous and collagenous matrix of fibroblasts mimicking the tunica adventitia. Fibrin/fibrinogen, the printing materials they used, has not been not a typical material for 3D bioprinting due to its poor printability. Although they overcame the problematic printability of fibrinogen through their new printing technique, the printing process is complex and inefficient [29]. Therefore, to advance vascular engineering, there is a great need to efficiently biofabricate biomimetic blood vessels having crucial native-like architecture, structural integrity, and biological functions [20,30,31,32]. 

To address the vascularization issue, we developed a novel strategy that employs biomaterials with high biocompatibility and a custom-built 3D bioprinting system to fabricate two types of blood vessels with highly ordered arrangements: blood vessels with a prevascularized cell-layer of endothelial cells in thick tissues and small-diameter blood vessels (diameter: 6 mm) with a tailored three-layer structure. In particular, small-diameter blood vessels with three layers can be designed flexibly with different wall thicknesses to mimic their natural various features to the highest degree. Moreover, the printed blood vessels can be used for pathological studies related to the blood and the construction of related pathological models for pharmaceutical/toxicological screening in vitro. For example, they can be used to establish a thrombus model to study the mechanism of thrombus development in vitro.

## 2. Materials and Methods

Matrix formulations of decellularized extracellular matrix (dECM): The preparation of dECM refers to the method introduced by Falguni Pati et al. It was prepared by mincing porcine cartilage followed by its placement into a hypotonic Tris-HCl buffer solution (10 mM Tris-HCl, pH 8.0) that underwent six cycles of freezing (at 80 °C) and thawing (at 37 °C). The cartilage slurry was homogenized and treated with 0.25% trypsin in PBS (Phosphate buffer saline) for 24 h at 37 °C with vigorous agitation. The trypsin solution was refreshed every 4 h. The trypsinized cartilage slurry was washed with a hypertonic buffer solution (1.5 M NaCl in 50 mM Tris-HCl, pH 7.6) and treated with nuclease solution (50 U/mL DNase and 1 U/mL RNase in 10 mM Tris-HCl, pH 7.5) with gentle agitation at 37 °C for 4 h. To remove all enzymes, the enzyme-treated cartilage slurry was washed with a hypotonic Tris-HCl solution for 20 h followed by treatment with 1% Triton X-100 solution for 24 h. The decellularized cartilage tissue was washed for 3 days to remove all detergent [33].

Ink formulations: The bioprintable supporting material Pluronic F127 was prepared according to the method introduced by David B. Kolesky et al. Specifically, 40 wt % Pluronic F127 (Sigma) in deionized, ultrafiltrated (DIUF) water was homogenized using a Thinky mixer until the powder was fully dissolved, and subsequently stored at 4 °C. The ink was loaded in a syringe at 4 °C and any air bubbles were removed prior to use [28].

A silicone ink, composed of a two-part silicone elastomer (SE1700; Dow Chemical) with a 10:0.6 base-to-catalyst ratio (by weight), was used to print a supporting scaffold used in the construction of small-diameter blood vessels. It was homogenized using a mixer (2000 rmp speed, AE-310, Thinky Corporation, Beijing, China) and printed within 2 h of mixing [26].

Rheological characterization: Ink rheology was measured using a controlled rotating rheometer (MCR302, Anton Paar, Graz, Austria) with a 25-mm diameter plate geometry. To assess the viscosity of dECM/PF127 pre-gels, steady shear sweep analysis was performed at a constant temperature of 4 °C. To assess the viscosity of SE1700 pre-gels, steady shear sweep analysis was performed at a constant temperature of 45 °C. Temperature sweeps, used to study the gelation kinetics, were performed over a range of 1 °C to 40 °C (dECM and Pluronic F127) or 10 °C to 80 °C (SE1700). For temperature sweeps, the modulus was measured at each temperature. Dynamic frequency sweep analysis was conducted to measure the frequency-dependent storage (G′) and loss (G″) moduli of dECM/PF127 (after incubation for 10 min at 37 °C) and SE1700 (after incubation for 10 min at 45 °C) gels in the range of 0.01–100 rad∙s^–1^ at 1% strain. In addition, at 80 °C, SE1700 pre-gels were held for 20 min to measure the time-dependent storage (G′) and loss (G″) moduli. All measurements were conducted in triplicate.

Cell culture and maintenance: Human dermal fibroblasts–neonatal (HDF-n) cells were cultured in fibroblast medium (FM) with 10% fetal bovine serum (FBS; Cat. No. 0010), 1% fibroblast growth supplement (FGS; Cat. No. 2352), 100 U/mL penicillin, and 100 μg/mL streptomycin (Invitrogen). Human aortic vascular smooth muscle cells (HA-VSMCs) were cultured in Dulbecco’s modified Eagle’s medium (DMEM) (Invitrogen) with 10% fetal bovine serum (FBS; HyClone), 100 U/mL penicillin, and 100 μg/mL streptomycin (Invitrogen). Human umbilical vein endothelial cells (HUVECs) were cultured in RPMI (HyClone) with 10% fetal bovine serum (FBS; HyClone), 100 U/mL penicillin, and 100 μg/mL streptomycin (Invitrogen). The cells were cultured in an incubator at 37 °C with an atmosphere of 5% CO_2_. The cells were subcultured using trypsin (0.25%; Invitrogen) upon reaching approximately 80% confluence. The culture medium was changed every 2–3 days. MDA-MB-231, breast cancer cells, from American Type Culture Collection (ATCC) were cultured in Leibovitz L 15 Medium (Life Technologies Corporation, Shanghai, China) supplemented with 10% fetal bovine serum, penicillin (100 units/mL), and streptomycin (100 μg/mL).

Fluorescent transfection: After 12–16 h of culture time in the Petri dish of cells, the medium was removed and a mixed liquid of 1 mL of GFP/RFP (green/red fluorescent protein) marker liquid, 3 mL of fresh culture medium, and 4 μL of polybrene was added into the dish. Then, at intervals of 15 to 30 min, the marked cell cultures were gently shaken, so that a better effect could be achieved. After 2 h, 24 h, and 48 h, 4 mL of fresh culture medium replaced the previous medium. Then, the successful fluorescent transfection was observed using a fluorescence microscope. 

Endothelialization of vascular networks: After the removal of the fugitive ink from a structure, 100–400 μL of the HUVEC suspension (5 × 10^6^ cells mL^−1^) was injected into hollow channels via a pipette. Both ends of the channel were sealed. Next, the structure was cultured in an incubator to facilitate HUVEC adhesion to the channels. The structure was flipped, and the HUVEC suspension was renewed regularly to form an intact endothelium with circumferential seeding of cells.

Imaging and analysis: All fluorescence images were obtained using a fluorescence microscope (X81, Olympus, Tokyo, Japan) integrated with a charge-coupled device (CCD) camera (Olympus XM10T, Hamamatsu ORCA-R2, Tokyo, Japan) and control software (Xcellence Imaging Software, version number, Tokyo, Japan). Fluorescent dyes were adopted for the improved visualization of MDA-MB-231 (green), HDF-n cells (red), HA-VSMCs (green), and HUVECs (red). Software Image-Pro Plus 6.0 (Media Cybernetics, Silver Spring, Rockville, MD, USA) and Origin 8.0 (OriginLab Corporation, Northampton, MA, USA) were employed to perform the image analysis and data statistical analysis, respectively. Data are presented as means ± standard deviations of the mean (SD).

Fabrication of supporting structures: Supporting structures were created using a custom-built extrusion-based 3D bioprinter (using nozzles that directly extrude materials onto a fabrication platform). This 3D bioprinter consists of a multi-nozzle system and a three-axis, motion-controlled platform with a volume of 150 mm × 40 mm × 125 mm [34]. The silicone ink was housed in a 3-mL syringe and was bioprinted on a glass plate through a tapered 150-μm plastic needle. A structure with inner and outer concentric walls was printed. Baking for 1 h at 80 °C in the oven (Hengyi, Shanghai, China) was followed by laser drilling by a laser perforator (Hans Laser, Shenzhen, China). The pore diameter was 0.5 mm with a 0.5-mm pitch, allowing cells and nutrients to migrate freely, which helps improve intercellular communication and material exchange between cells and the matrix. The supporting scaffold was immersed in polylysine solution at 37 °C for 2 h to promote the adhesion of cells to its interior and surface.

3D bioprinting of blood vessels with a prevascularized cell-layer of HUVECs: We adopted a four-nozzle 3D bioprinter, with dECM and Pluronic F127 as printing materials, to print a thick structure with multilevel vascular channels using a layer-by-layer printing method. Pluronic F127 is a temperature-sensitive phase change material and can be dissolved in deionized water at 37 °C [28]. At the normal temperature (37 °C), it exists in a gel state, but remains in a sol state at 4 °C [35]. The dECM also has phase-transition temperature-sensitive properties and is similar to Pluronic F127 in this case. The dECM was printed at 4 °C, and the printed structure was incubated for 30 min~1 h at 37 °C to form a stable gel state. Next, the thick structure was transformed into deionized water to remove Pluronic F127 (while Pluronic F127 is soluble in water, the dECM in a gel state is insoluble in water). According to the features of Pluronic F127 and dECM, we designed this facile strategy for bioprinting thick tissues using multilevel perfusable hollow channels before the formation of an intact endothelium. 

**Figure 1:**
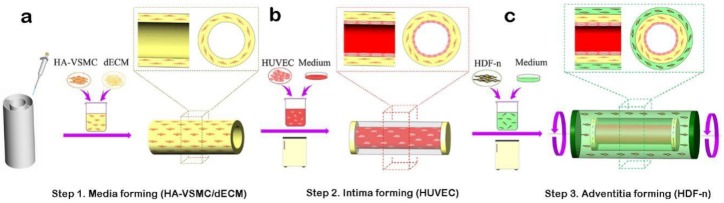
Schematic illustration of the fabrication process of three-dimensional (3D) arteriovenous structures with three layers: intima, media, and adventitia. (**a**) Addition of the decellularized extracellular matrix (dECM) with human aortic vascular smooth muscle cells (HA-VSMCs) between the inner layer and outer layer of the support structure. (**b**) A human umbilical vein endothelial cells (HUVEC) suspension (5 × 10^6^ cells mL^−1^) was injected into hollow channels via a pipette. (**c**) Construction of the adventitia layer by immersing the structure into a human dermal fibroblasts–neonatal (HDF-n) suspension (5 × 10^6^ cells mL^−1^) on the basis of the first two layers.

Construction of small-diameter blood vessels: First, to construct the media, after mixing with HA-VSMCs, dECM was added between the inner and outer circular walls of a supporting scaffold. At 37 °C, it gradually changed into a gel, and the entire process lasted approximately 0.5 h (Figure 1a). Second, to construct the intima, a HUVEC suspension (5 × 10^6^ cells mL^−1^) was perfused into the inner channel of a supporting scaffold, and then the scaffold was cultured in an incubator. The structure was reversed by 90° every hour, and the HUVEC suspension was renewed at the same time (Figure 1b). Third, to construct the adventitia, the HDF-n suspension (5 × 10^6^ cells mL^−1^) was used to immerse the abovementioned structure. The structure was flipped, and the HDF-n suspension was renewed regularly. HDF-n cells gradually attached to the outer wall of the supporting scaffold. The whole structure was then transferred to an incubator (Thermo Fisher Scientific, Waltham, MA, USA) for follow-up culture. The whole process took approximately 36 h. Thus, a small-diameter blood vessel with three layers (intima, media, and adventitia) was completely constructed (Figure 1c). 

## 3. Results

### 3.1. The Rheological Properties of the Bioink 

**Figure 2:**
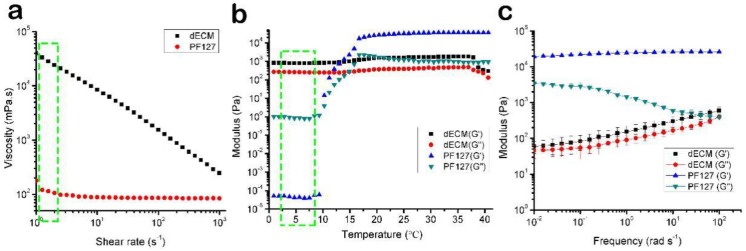
(**a**) Rheological properties of the dECM and PF127 viscosity at 4 °C. (**b**) Gelation kinetics from 1 °C to 40 °C (initial temperature 1 °C, increment of 1 °C min^−1^, reaching up to 40 °C.). (**c**) Dynamic modulus with varying frequency at 37 °C. All experiments were performed in triplicate. Error bars represent the SD.

**Figure 3:**
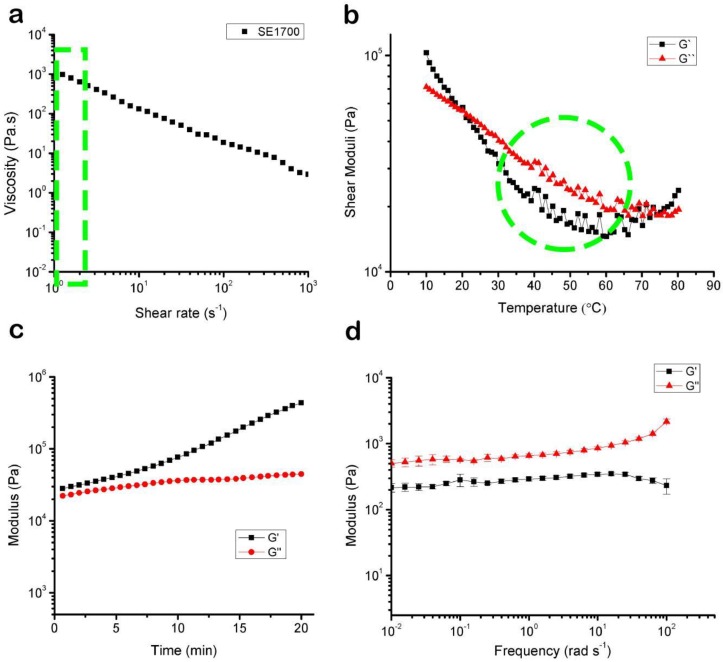
(**a**) Rheological properties of the SE1700 viscosity at 45 °C. (**b**) Gelation kinetics from 10 °C to 80 °C (initial temperature 10 °C, increment of 1 °C min^−1^, reaching up to 80 °C). (**c**) The time-dependent shear storage (G′) and loss (G″) moduli were held at 80 °C for 20 min. (**d**) Dynamic modulus with varying frequency at 45 °C. All experiments were performed in triplicate. Error bars represent the SD.

Rheological properties of dECM pre-gels and Pluronic F127 were measured to evaluate their flow behavior, as shown in Figure 2. dECM showed shear thinning behavior in the measured shear rate range (Figure 2a). We exploited the behavior of 40 wt % Pluronic F127, which exhibits a strong shear-thinning response when the applied shear stress exceeds the shear yield stress [28] during the printing process. The generated shear rate in our printing method through a 250-μm nozzle was in the range of 0.72–2.36 s^−1^.The viscosities of the bioinks at a shear rate of 2 s^−1^ were 24.27 Pa∙s (dECM) and 0.11 Pa∙s when measured at 4 °C. Concerning the gelation kinetics of the dECM/PF127 at varying temperatures starting from 1 to 40 °C with a temperature ramp of 1 °C, we found that the modulus of PF127 was almost 2 × 10^4^ Pa at 25 °C (Figure 2b). Below 10 °C, liquification occurred and the modulus of PF127 decreased by several orders of magnitude (Figure 2b). Dynamic modulus with varying frequency at 37 °C (Figure 2c). The rheological properties of SE1700 are shown in Figure 3.

### 3.2. Printed Instances of the Custom Multi-Nozzle 3D Bioprinting System

**Figure 4:**
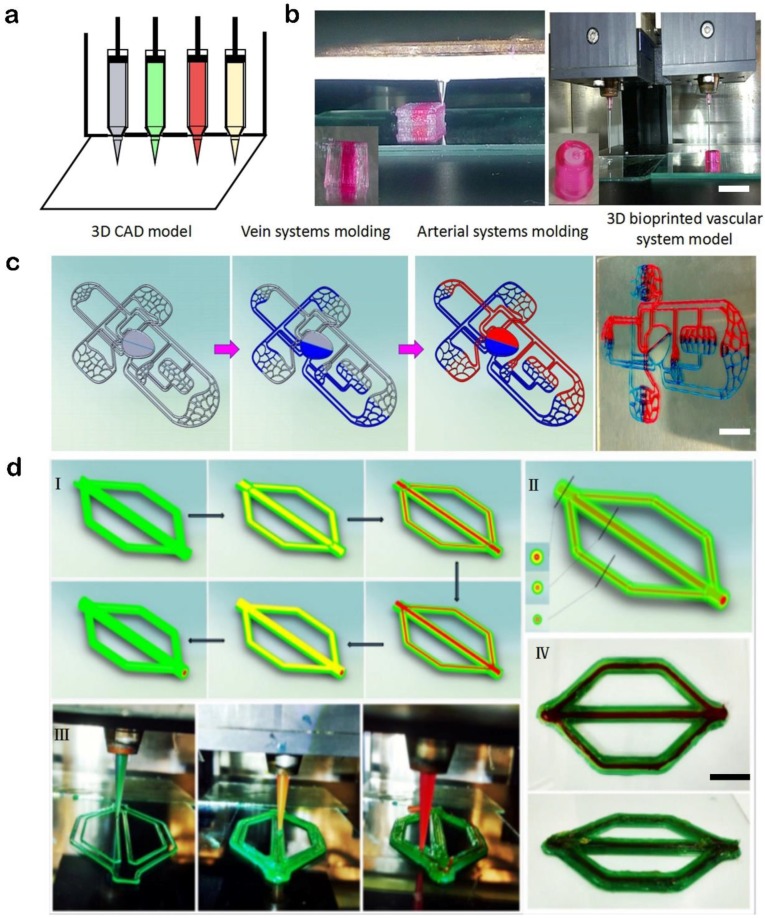
(**a**) Schematic diagram showing four independent nozzles of the 3D bioprinter. (**b**) Fabrication of tissue structures using a multiple nozzle system (Scale bar: 2 cm). (**c**) Model formation schematics of the vascular system containing arteries and veins and the actual vascular system model (Scale bar: 2 cm). (**d**) A specially designed vascular model with three layers of structure (Scale bar: 1 cm).

Different kinds of 3D structures (Figure 4) have been printed using a custom-built 3D bioprinter developed by our group. Inks were housed in 3-mL syringes mounted to independently controlled extrusion nozzles. Needles and syringes were connected via Luer locks. Different kinds of needles were used, including stainless steel needles (from 150 µm to 500 µm in diameter) and tapered plastic needles (from 150 µm to 500 µm in diameter). With this versatile 3D bioprinting system, we could biofabricate 3D structures with a wide range of bioinks, such as dECM and Pluronic F127.

### 3.3. Manufacture of Blood Vessels with a Prevascularized Cell-Layer of HUVECs in Thick Tissues

As the simplest blood vessels in the human body, blood vessels with a prevascularized cell-layer of HUVECs contain only a complete vascular wall, but they are still very important carriers for oxygen absorption, nutrient delivery, and metabolism in vivo. Therefore, constructing this type of vessel is vital to meet the long-term culture needs of tissue culture in vitro without the production of necrotic sites during this period. 

**Figure 5:**
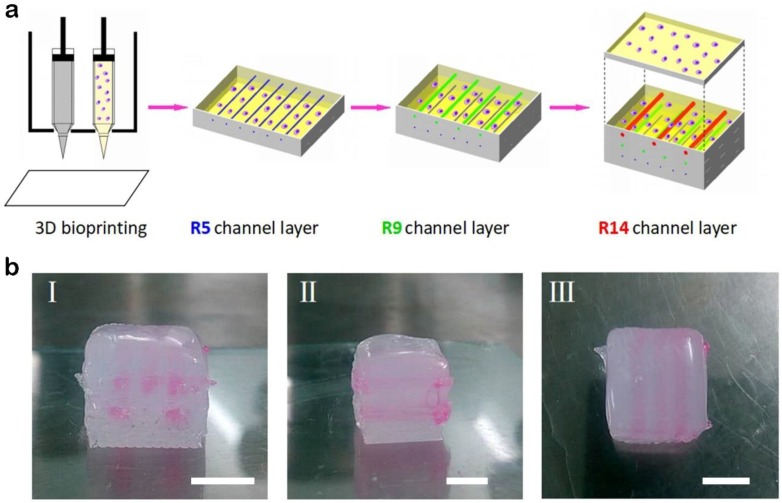
A multilevel channel structure printed by this method. (**a**) Schematic illustration showing the procedure of biofabricating thick tissues with hollow channels of three different diameters: broad channels (radius: 1.4 mm), medium channels (radius: 0.9 mm), and narrow channels (radius: 0.5 mm). (**b**) Actual pictures of a multilevel channel structure: (I) front view, (II) side view, (III) top view. (Scale bar: 5 mm).

In the present study, we used the abovementioned 3D bioprinting system to fabricate thick tissues with multilevel vascular structures. A thick tissue could be divided into three parts. The first is the bottom part with external Pluronic F127 as a support structure and dECM loading with MDA-MB-231 cells inside. The middle part, with internal Pluronic F127 printed as a sacrificial material and external Pluronic F127 printed as a support structure, as well as dECM loading with cells printed inside, includes multilevel channels, large channels (red, hydraulic diameter: 1.4 mm), medium channels (green, hydraulic diameter: 0.9 mm), and small channels (blue, hydraulic diameter: 0.5 mm). The top part is similar to the bottom part. This printing process is shown in Figure 5a, and actual pictures of a structure fabricated with this method are shown in Figure 5b.

**Figure 6:**
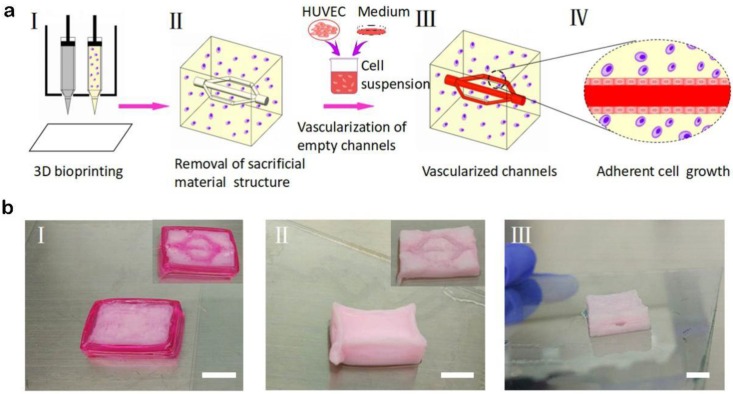
A multibranch channel structure printed by this method. (**a**) Schematic illustration showing the procedure of biofabricating thick tissues with multibranch channels. (I) In the 3D bioprinting process, Pluronic F127 as the fugitive ink and cell-laden dECM as the matrix were bioprinted. (II) After the tissue was immersed in a constant-temperature bath for 1 h, Pluronic F127 was liquefied and removed. Next, perfusable hollow channels were formed, and (III) endothelialized afterwards. (IV) The suspension of HUVECs was perfused to the inside of the hollow channel to form the intact endothelial layer. (**b**) Actual pictures of a multilevel channel structure. (Scale bars: 1 cm).

Using the method mentioned above, a thick structure with multibranch vascular channels was also constructed (Figure 6a, I and II). In particular, for this structure, the printing processes of the bottom and top parts were similar to those of structures with multilevel vascular channels, but the middle part contained multibranch channels instead of straight channels. The multibranch channels made it possible for the HUVEC suspension to flow from the entrance and then through the branch vessels to eventually converge to the exit. In fact, depending on the flexibility of the computer-aided design, different shapes of vascular structures could be designed and printed with this strategy.

**Figure 7:**
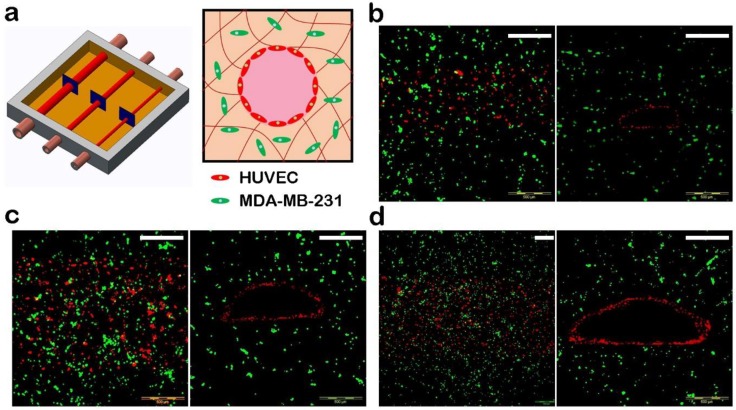
Three-dimensional vascularized tissues. (**a**) Schematic depicting a HUVEC-lined vascular channel. (**b**–**d**) Confocal microscopy images of the vascular network after 7 days; HUVEC (red), MDA-MB-231 (green). Chanel hydraulic diameters: (b) 1.4 mm, (c) 0.9 mm, and (d) 0.5 mm. (Scale bars: 500 μm).

After printing, the printed structure was placed in a water bath in which the water temperature was 37 °C, and the Pluronic F127 in both the internal and external areas was removed, leaving behind a pervasive vascular network. The multilevel hollow channels were then perfused with the HUVEC suspension (5 × 10^6^ cells mL^−1^) to form blood vessels with an intact endothelium (Figure 6). Confocal microscopy images of the vascular network are shown in Figure 7.

### 3.4. Bioprinting of Supporting Scaffolds and Mechanical Performance Testing

**Figure 8:**
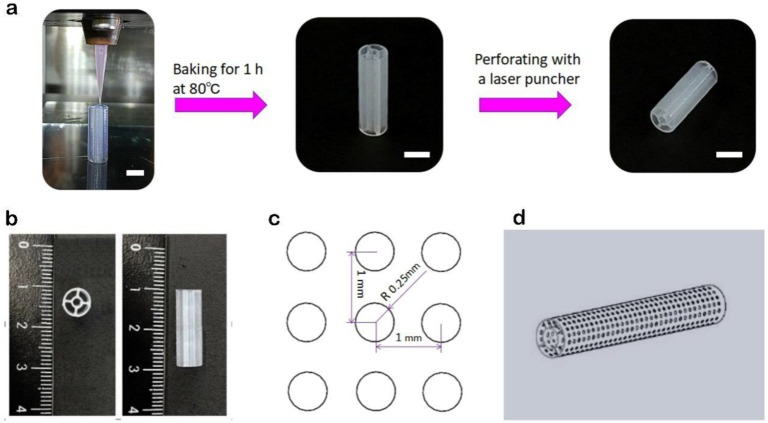
Construction processes of the supporting structure. (**a**) 3D bioprinting according to a CAD (computer-aided design) model, baking, molding, and laser drilling. (Scale bar: 5 mm). (**b**) Inner and outer cylindrical supporting structure. (**c**) Size and spacing of laser drilling. (**d**) A pattern diagram of the supporting structure with micropores.

**Figure 9:**
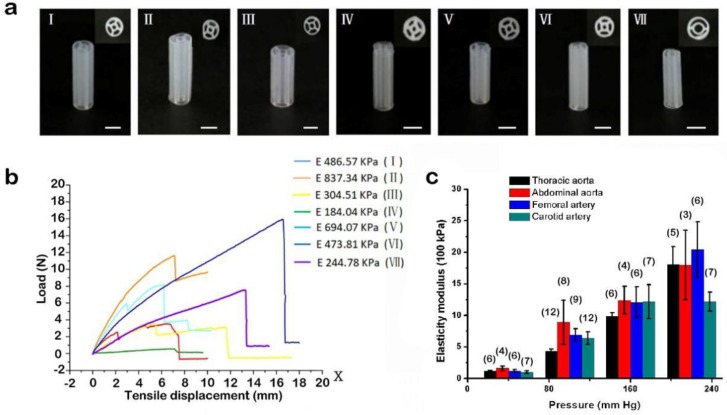
Mechanical properties of the supporting scaffold. (**a**) Varying ratios of SE1700 to the catalyst (For I~IV, the ratios were 10:1, 10:0.6, 10:0.5, and 10:0.3, respectively; their wall thicknesses were the same, i.e., approximately 250 μm. For V~VII, the wall thicknesses were different—250 μm, 200 μm, and 150 μm, respectively—and their ratios of SE1700 to the catalyst were the same—10:0.6 μm). (Scale bar: 5 mm). (**b**) Load and tensile displacement curves of the samples (I~VII). (**c**) Mean values for the static elastic modulus, with the number of measurements (specimens) shown in brackets [36].

A good choice for supporting scaffolds is silicone ink. Silicone ink is known for its non-toxicity and sufficient mechanical strength. A structure with inner and outer concentric walls was printed using silicone ink, as shown in Figure 8. Moreover, its mechanical strength varies according to the ratio of SE1700 to the catalyst and wall thickness (Figure 9a). For the manufacture of the arteriovenous structure, SE1700 mixed with a catalyst at a ratio of 10:0.6 (by weight) was adopted, and the wall thickness was approximately 150 μm. At this ratio, the elastic modulus of the supporting scaffolds was 244.78 KPa (Figure 9b), which is closest to the elastic modulus of a real thoracic aorta (Figure 9c) [36]. Therefore, SE1700 with a 10:0.6 base-to-catalyst ratio was selected as the supporting material to subsequently construct small-diameter blood vessels.

### 3.5. Manufacture of Small-Diameter Blood Vessels 

Here, we provide a widely applicable construction method that can be used to construct specific artery-like or vein-like structures by changing the corresponding parameters (e.g., the ratio of SE1700 to the catalyst and the wall thickness of the supporting scaffolds).

To meet the mechanical strength requirements, the elastic properties of the printed structure were enhanced by the introduction of supporting scaffolds. For the matrix material, dECM was adopted because of its good biocompatibility. Its natural components can provide the nutrients required for vascular cells and can fulfill cell growth and proliferation requirements.

**Figure 10:**
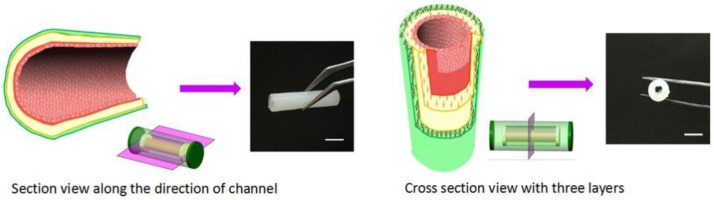
Schematic illustration of the three-layered arteriovenous structure and its transverse and longitudinal sectional view. (Scale bar: 5 mm).

**Figure 11:**
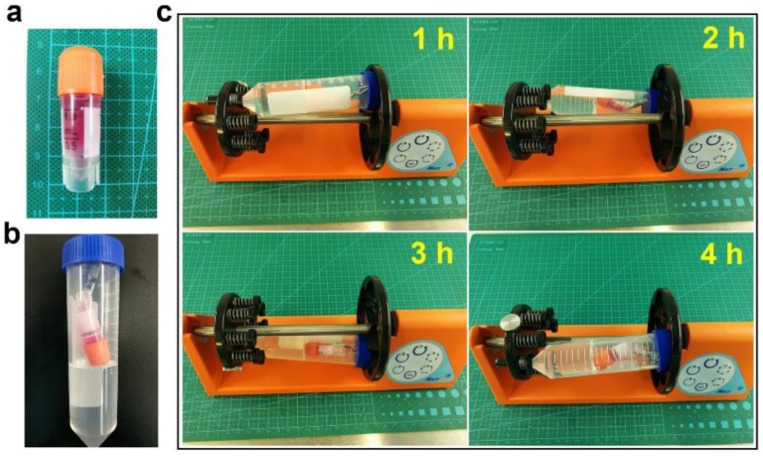
The rotating stage. (**a**) An arteriovenous structure in a 1-mL frozen storage tube, filled with cells and medium. (**b**) The frozen storage tube was put into the centrifuge tube and then settled. (**c**) The rotating process.

**Figure 12:**
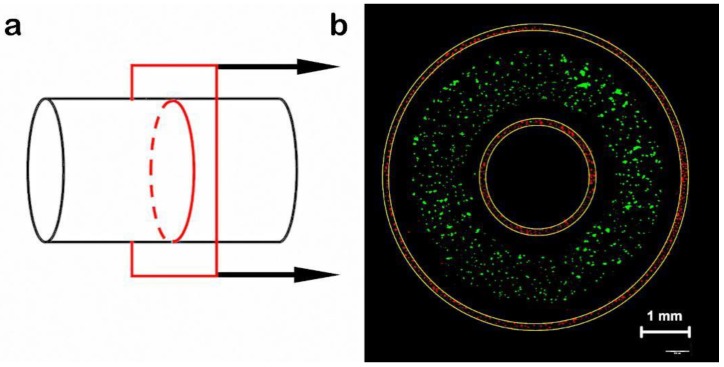
(**a**) Schematic views of cross section. (**b**) An arteriovenous structure with three layers, in which the outer red, green and inner red parts correspond to the HDF-n-laden adventitia, HA-VSMC-laden media, and HUVEC-laden intima, respectively. Actual pictures of the 3D arteriovenous construct were acquired using three fluorescent labels (Scale bar: 1 mm).

The transverse and longitudinal sectional views of an arteriovenous structure are shown in Figure 10, with the schematic diagram on the left and a physical picture on the right. The rotating process is shown in Figure 11. The intima was comprised of HUVECs (transfected with red fluorescent protein (RFP)). The media consisted of dECM loaded with HA-VSMCs (transfected with green fluorescent protein (GFP)), and the adventitia was mainly comprised of dECM and HDF-n (transfected with red fluorescent protein (RFP)) (Figure 12). 

## 4. Discussion

Compared to other methods of tissue engineering, 3D bioprinting is a rapid and highly efficient method that can accurately realize the co-printing of multi- cells and multi- materials. 

Multiple studies have already proven that complex 3D structures are printable and usable [20,26,37,38,39].

Extrusion methods, using a multi-nozzle system to bioprint multi-materials simultaneously, can biofabricate 3D cell-laden engineering tissues with improved structural integrity [30]. Based on the above context, the 3D cell printer developed by our group adopts an extrusion-based system and is equipped with four nozzles. Each nozzle contains an independent temperature control system. The printing temperature can be controlled from 0 °C to 60 °C. Additionally, various syringe needles can be used flexibly for different applications—e.g., stainless-steel needles (150 µm to 500 µm in diameter), ordinary plastic needles, and tapered plastic needles (150 µm to 500 µm in diameter). By optimizing the printing conditions, such as using a high-resolution, efficient bioinks, an optimal printing speed and an appropriate ambient temperature, our 3D bioprinter can conveniently produce highly controlled cell-laden structures for applications in engineering tissue reconstruction and in vitro disease modeling for pathological study. We demonstrated that the printed structures with intricate solid geometries have exceptional shape fidelity. Known methods incorporating cells in hydrogels during printing provide high stiffness but are impaired during cells growth and migration, making it difficult to form vascularized channels [40,41]. Direct 3D bioprinting cannot print a cell-layer adhered in a vascularized channel within tissue constructs [12,20].

Moreover, we showed that multiple biomaterials, Pluronic F127 and dECM, could be used to biofabricate various tissue constructs with crucial native-like architectures and adequate mechanical stability [28,33]. Furthermore, our 3D bioprinting method is flexible with a powerful software system such that heterogeneous hybrid architectures can be generated and modified freely by just writing and revising an initial program. It can also be suitable for controlling cell density within printed structures by setting the required overprinting number in corresponding programs. In addition, our custom-built 3D bioprinting platform could be further combined with other deposition technologies for advanced tissue engineering applications.

In general, our 3D bio-printing technology is also a promising tool to construct engineering blood vessels for the gradual substitution of traditional real tissue-based pathological analysis and drug screening. Successful biomimetic blood vessels must meet certain requirements, such as native-like viscoelasticity, appropriate remodeling responses, and vasoactivity [42]. Current strategies for blood vessel engineering are relatively unsatisfactory and fail to meet one or more of these requirements. Based on our custom-built 3D bioprinting system, a versatile strategy was developed to biofabricate prevascularized cell-layer blood vessels in thick tissues and small-diameter blood vessel substitutes with high biocompatibility (suitable for cell growth, migration, and reproduction) and mechanical properties (replicating in vivo architectural features). For prevascularized cell-layer blood vessels in thick tissues, traditional vascularized methods often adopt top-down approaches in which cells are seeded later on bioprinted scaffolds. The drawback of these top-down approaches is that it is difficult for cells to maintain their biological function in their seeded locations. In this study, a bottom-up strategy was developed to biofabricate prevascularized cell-layer blood vessels in thick tissues by using cell-laden dECM as a matrix material and Pluronic F127 as a sacrificial material. These vascularized thick tissues with multilevel or multibranch channel architectures exhibited improved stability and high biocompatibility, showing great promise in engineering tissue prevascularization and the further study of tissue physiology and function. Compared to direct formation methods, the developed strategy for the reconstruction of small-diameter blood vessels is versatile. A supporting scaffold (SE1700) with a double-layer circular structure was 3D bioprinted first, prior to the formation of the media, intima, and adventitia. Supporting scaffolds could provide sufficient mechanical strength to maintain the structural integrity and could be printed with different wall thicknesses and transverse sizes, which means that they can be flexibly applied to different aperture classes of arteriovenous structures. The constructed small-diameter blood vessels can be used for the construction of pathological models in vitro and for the elucidation of vascular-related cell biology mechanisms, disease-developing mechanisms, and drug screening. For example, they can be used to construct a thrombus model to study thrombosis fibrosis development in the blood when the vascular endothelial layer integrity is damaged, which leads to the migration of fibroblasts.

Future studies will include how prevascularized cell-layer blood vessels and small-diameter blood vessels can be joined together to form a complete vascular network. This vascular network will be very similar in structure and function to blood vessels in the body. In addition, the double-layered supporting scaffold (SE1700) with laser micropores has good permeability and is appropriate for material exchange and cell migration on both sides. It can also be highly suitable for the construction of co-culture models of different kinds of cells to study cell-matrix and cell-cell interactions. 

## 5. Conclusions 

In this study, to accommodate the diverse structures of human blood vessels, prevascularized cell-layer blood vessels and small-diameter blood vessels were constructed in different ways. Specifically, to construct prevascularized cell-layer blood vessels, a sacrificial material (Pluronic F127) was removed to form multilevel and multibranch hollow channels. To construct small-diameter blood vessels, an inner and outer double-layered SE1700 supporting structure was printed to improve the mechanical properties of the structure. In addition, considering that dECM and SE1700 have good biocompatibility and provide sufficient mechanical strength, similar to that of the natural aorta, the constructed small-diameter blood vessels are highly biocompatible. After culture in vitro for 48 h, fluorescent images revealed that cells maintained their viability and that the sample maintained structural integrity. Finally, there are extensive application prospects for constructing these vascular structures in the future, such as blood pathological studies and the construction of vascular-related pathological models for pharmaceutical/toxicological screening in vitro and prevascularization for engineering thick tissues.
